# Trends in the Prescription of Benzodiazepine Receptor Agonists from 2009 to 2020: A Retrospective Study Using Electronic Healthcare Record Data of a University Hospital in Japan

**DOI:** 10.3390/healthcare9121724

**Published:** 2021-12-13

**Authors:** Tasuku Okui, Jinsang Park, Akie Hirata, Naoki Nakashima

**Affiliations:** 1 Medical Information Center, Kyushu University Hospital, Fukuoka 812-8582, Japan; hirata.akie.006@m.kyushu-u.ac.jp (A.H.); nakashima.naoki.351@m.kyushu-u.ac.jp (N.N.); 2 Department of Pharmaceutical Sciences, International University of Health and Welfare, Fukuoka 831-8501, Japan; park21@iuhw.ac.jp

**Keywords:** hypnotics, anxiolytics, benzodiazepines, prescriptions, Japan, electronic health records

## Abstract

In recent years, the prescription trends of benzodiazepine receptor agonists (BZRAs) have not been investigated in Japan despite the publication of guidelines that promote cautious use of BZRAs. The prescription trend of BZRAs was assessed using the electronic healthcare records data of a University Hospital in Japan. The data from April 2009 to March 2021 were used. The following three types of outcomes were set: the proportion of patients who were prescribed with BZRAs within those prescribed hypnotics or anxiolytics; the mean number of the types of prescribed BZRAs, and the mean average daily doses of BZRAs. The same analysis was conducted for benzodiazepines (BZDs) and non-benzodiazepines (Z-drugs). As a result, we found that the proportions of patients prescribed BZRAs within those prescribed hypnotics or anxiolytics began to decrease, particularly from 2015 for patients aged <75 years and those aged ≥75 years. Further, the degree of decrease was larger in patients aged ≥75 years. The proportion for BZDs decreased particularly in the study period, and the proportion for Z-drugs also began to decrease approximately from 2016 in patients aged ≥75 years. The results suggest a possibility that guidelines affected the decreased prescriptions of BZRAs.

## 1. Introduction

Polypharmacy of psychotropic drugs (i.e., adverse effects due to the simultaneous consumption of multiple kinds of psychotropic drugs) has been gaining attention in recent years in Japan [1], and some public policies were implemented in the previous decade to reduce the multi-drug use of psychotropic drugs [2]. Among psychotropic drugs, benzodiazepine receptor agonists (BZRAs) are known to have some adverse effects. BZRAs induce adverse effects, such as delirium, falls, and decline in cognitive functions [3,4,5]. BZRAs are frequently prescribed in clinical practice in Japan [6,7], while some recent guidelines have indicated the risk of BZRAs, particularly in the elderly in Japan [8,9]. Guidelines for Medical Treatment and its Safety in the Elderly 2015, published by the Japan Geriatrics Society, designates BZRAs as a type of drug that needs to be prescribed cautiously for the elderly [8]. Moreover, following the revision of the medical fee system in 2018, the reimbursement of medical fees by a “healthcare bill check and payment organization” began to be reduced when a BZRA was prescribed as an anxiolytic or hypnotic with same usage and dosage continuously for >1 year [2].

Several previous studies have investigated the actual conditions of BZRA prescriptions in Japan [1,6,7,10,11], and the actual status of the long-term use or characteristics of patients using BZRAs has been revealed. Specifically, BZRAs were shown as the most common hypnotic drugs prescribed as the first line of treatment to patients in Japan [7], and 9% of new benzodiazepine (BZD) users were given more than eight months of prescription [11]. Additionally, long-term BZD drug prescription occurred more frequently in older patients [6], and the psychiatrist-prescriber was found to be a predictor of long-term BZD use [10]. However, only one study has investigated the prescription trends of BZRAs during the previous decade [1]. A previous study showed the prescription trend from 2012 to 2017 and indicated that proportions of patients prescribed ≥3 BZRAs did not decrease during the analyzed periods in Japan [1]. However, a medical fee reduction is not applied even if one BZRA is prescribed as an anxiolytic and two BZRAs are prescribed as hypnotics, and it might not be surprising that the proportion of patients prescribed ≥3 BZRAs has not decreased over the years. In addition, previous studies in Japan did not investigate the trends in the proportion of patients prescribed BZRAs among those prescribed anxiolytics or hypnotics in recent years. If other types of anxiolytics or hypnotics were more actively prescribed in recent years, it is possible that the proportions were reduced over the years. Moreover, to our knowledge, the prescription trends after 2018 have not been investigated in previous studies, and an effect of the recent medical fee revision on prescriptions of BZRAs has not been revealed. Furthermore, the long-term prescription trend of BZRAs for patients aged ≥75 years in Japan is unknown. The adverse effects of BZRAs are known to particularly occur in the elderly [3,4,5], and as per major guidelines, prescriptions for elderly persons (patients aged ≥75 years) should be made carefully [8,9]. Therefore, it is important to investigate the prescription trends in patients aged ≥75 years. Apart from these points, BZRAs are classified into BZDs and Z-drugs (non-benzodiazepines) [12,13,14], and the long-term prescription trends for each type of BZRA, specifically BZDs and Z-drugs, have not been investigated in previous studies. The prescription trends of these types of drugs differed depending on the types of drugs in Spain [15], where BZRAs are one of the most commonly prescribed drugs for patients in polypharmacy [16]. This may also be true for Japan.

In this study, we aimed to investigate the prescription trends of BZRAs in recent decades using the electronic healthcare records (EHR) data of a University Hospital in Japan.

## 2. Materials and Methods

Data of the period from April 2009 to March 2021 from the EHR of Kyushu University Hospital in Japan were used. Every prescription conducted in the hospital in the analyzed periods was obtained, and data with anonymous patient ID, patient age, patient sex, prescription date, drug name, dosages, and administration periods for all the prescriptions were used. Patients who were prescribed anxiolytics or hypnotics at least one time in the target periods were included in the analysis, and those aged ≤19 year at the time when prescription was conducted were excluded from the analysis. BZRAs such as Alprazolam, Bromazepam, Brotizolam, Chlordiazepoxide, Clorazepate dipotassium, Clotiazepam, Cloxazolam, Diazepam, Estazolam, Ethyl loflazepate, Etizolam, Fludiazepam, Flunitrazepam, Flurazepam, Flutazolam, Flutoprazepam, Haloxazolam, Lorazepam, Lormetazepam, Medazepam, Mexazolam, Nimetazepam, Nitrazepam, Oxazolam, Prazepam, Quazepam, Rilmazafone, Triazolam, Tofisopam, Eszopiclone, Zopiclone, and Zolpidem were on sale in the analyzed periods in Japan. Among the BZRAs, the drugs (except for Nimetazepam, Prazepam, Haloxazolam) were prescribed in the Hospital in the analyzed periods. Eszopiclone, Zopiclone, and Zolpidem are Z-drugs, and the other drugs are BZDs.

We set three types of outcomes for this study, including the proportion of patients prescribed BZRAs within those prescribed hypnotics or anxiolytics in a month, the mean of number of the types of BZRAs prescribed to a patient in a month, and the mean of average daily doses of BZRAs (diazepam-equivalent mg/day) for a patient in 1 month. The proportion of patients prescribed with BZRAs within those prescribed with hypnotics or anxiolytics in a month was calculated to observe whether the proportion of BZRAs users decreased among users of hypnotics or anxiolytics over the years. The number of patients prescribed BZRAs and hypnotics or anxiolytics for each month was calculated, and the proportion of BZRAs users was calculated. This indicator does not evaluate whether the number of types of BZRAs prescribed to a BZRAs user decreased over the years, and we calculated the mean of number of BZRAs prescribed to a patient in a month. The number of types of BZRAs was counted for each patient in each month, and we calculated the mean value among BZRAs users for each month. The number of the types of drugs for patients who switched from one type of drug to another in a month was counted as two in a month. In addition to these indicators, we calculated the mean of average daily dose of BZRAs (diazepam-equivalent mg/day) for a patient in a month, as in a previous study to evaluate whether dosages decreased among BZRAs users [1]. We calculated the average daily doses of BZRAs for a patient in a month based on the total amount of dosages of BZRAs (diazepam-equivalent mg) and administration periods wherein the dosages were consumed. Then, the mean value of the average daily doses among patients in each month were calculated. Differences in the titers among BZRAs were considered when calculating the total amount of dosages, and Diazepam-equivalent doses were calculated for each patient in a month, as in previous studies [1,17]. The same analysis was also conducted for BZDs and Z-drugs. Changes in each outcome indicator were plotted by the types of drugs (BZRAs, BZDs, and Z-drugs).

Additionally, we calculated the monthly percent change (MPC) of an outcome value in the analyzed periods for each outcome. Guidelines for Medical Treatment and its Safety in the Elderly 2015 was published in December 2015 by the Japan Geriatrics Society, and it designates BZRAs as a type of drug that needs to be cautiously prescribed for the elderly. Moreover, the revision of the medical fee system on BZRAs was enforced in April 2018. Therefore, we divided the analyzed periods into three segments, i.e., April 2009–December 2015, December 2015–April 2018, and April 2018–March 2021, and we calculated the MPC for each period.

All the analyses were conducted as per two age groups, including those aged <75 years and those aged ≥75 years. We used the cut-off value of 75 years because the prescriptions particularly for elderly persons (patients aged ≥75 years) should be made carefully as per the major guidelines in Japan [8,9]. Therefore, verifying the difference in prescription trends between patients under 75 years and those aged ≥75 years is meaningful. All the statistical analyses were conducted using R version 3.6.3 (https://cran.r-project.org/bin/windows/base/old/3.6.3/ accessed on 9 December 2021).

This study was performed following the Declaration of Helsinki and was approved by the ethical committees of the Faculty of Medicine of Kyushu University (2021-76).

## 3. Results

The data of 67,756 patients were analyzed. Table 1 shows the annual characteristics of the patients who were prescribed anxiolytics or hypnotics. About 10,000 persons were prescribed anxiolytics or hypnotics at least once each year in the hospital. The proportion of patients who were prescribed BZRAs and BZDs decreased over the years.

**Figure 1:**
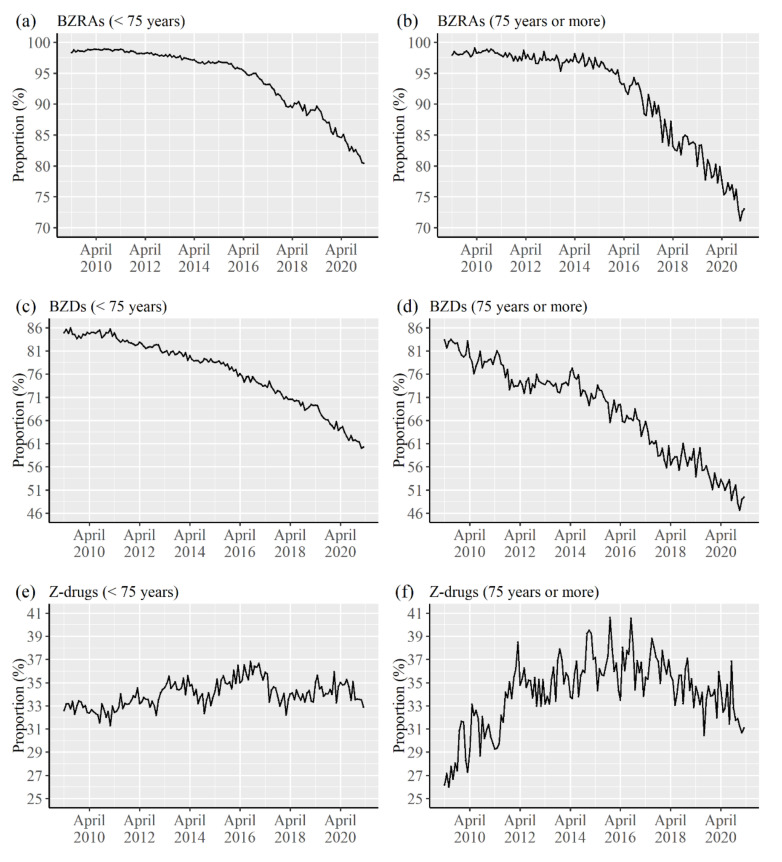
Trends of the proportion of patients prescribed drugs among those prescribed hypnotics or anxiolytics in a month by age group and type of drug. (**a**) Proportion for BZRAs in patients aged <75 years. (**b**) Proportion for BZRAs among patients aged ≥75 years. (**c**) Proportion for BZDs among patients aged <75 years. (**d**) Proportion for BZDs among patients aged ≥75 years. (**e**) Proportion for Z-drugs among patients aged <75 years. (**f**) Proportion for BZDs among patients aged ≥75 years.

Figure 1 shows the trends of the proportion of patients prescribed with the drugs within those prescribed hypnotics or anxiolytics in a month as per age groups and the types of drugs. The proportion of patients prescribed BZRAs began to particularly decrease from 2015, irrespective of the age groups, and the degree of the decrease was larger in those aged ≥75 years. The proportion for BZDs continuously decreased from 2009 for patients aged ≥75 years. However, the proportion for Z-drugs showed an increasing trend until about 2016 in both the age groups, and a decrease was observed subsequently for patients aged ≥75 years.

**Figure 2:**
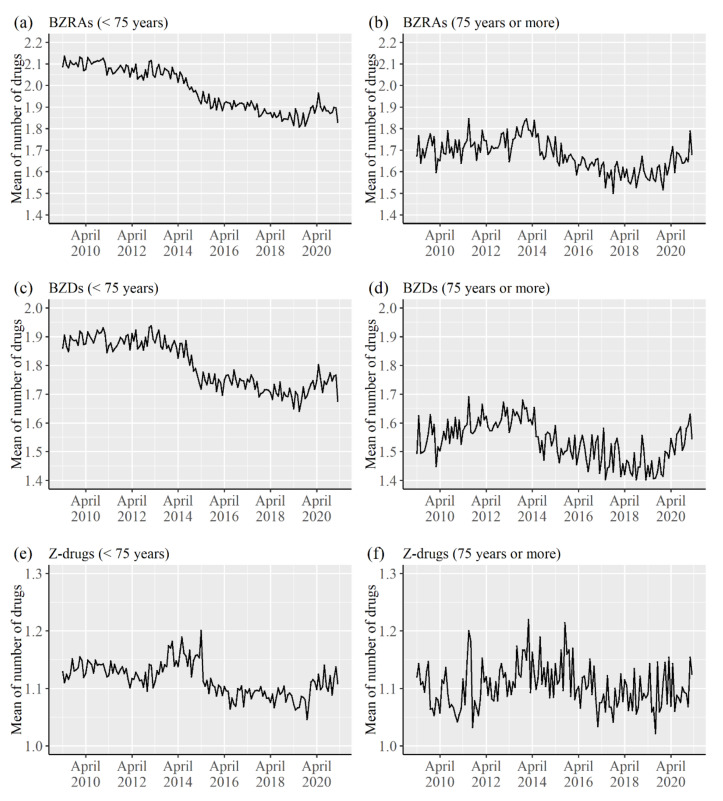
Trends of the mean of number of the types of drugs prescribed to a patient in a month as per the age group and type of drugs. (**a**) Mean for BZRAs in patients aged <75 years. (**b**) Mean for BZRAs among patients aged ≥75 years. (**c**) Mean for BZDs among patients aged <75 years. (**d**) Mean for BZDs among patients aged ≥75 years. (**e**) Mean for Z-drugs among patients aged <75 years. (**f**) Mean for BZDs among patients aged ≥75 years.

Figure 2 shows the trends of the mean of the number of types of drugs prescribed to a patient in a month as per the age group and type of drugs. A continuous decreasing trend was observed for BZRAs among patient aged <75 years. However, the mean value decreased from about 2014 in patients aged ≥75 years, while it showed an increasing trend in the previous few years. The trends for BZDs and Z-drugs were a little bit different among patients aged <75 years, while they were relatively similar among those aged ≥75 years.

**Figure 3:**
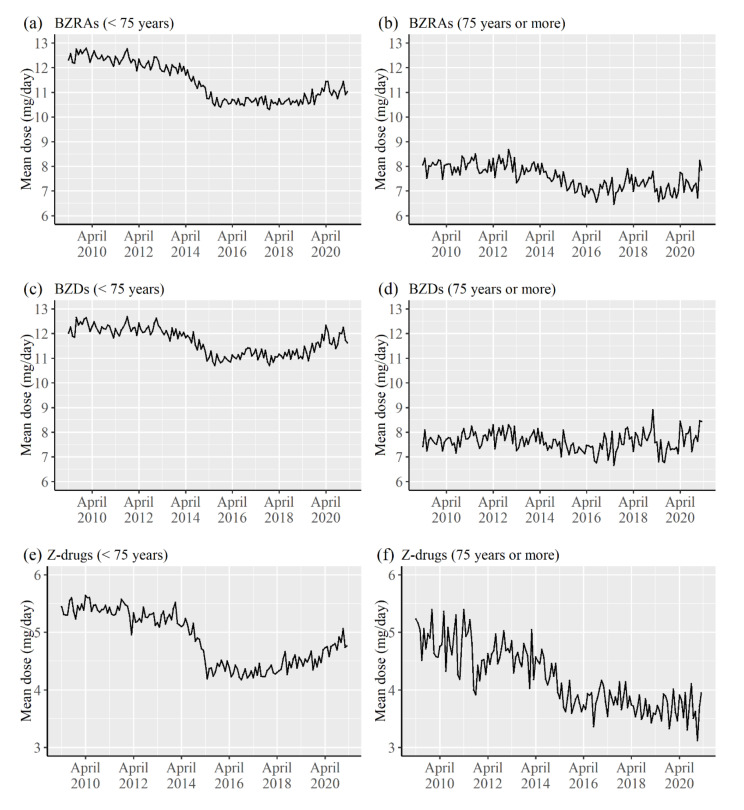
Trends of the mean of average daily doses (diazepam-equivalent mg/day) for a patient in a month by the age groups and the type of drug. (**a**) Mean for BZRAs in patients aged <75 years, (**b**) Mean for BZRAs among patients aged ≥75 years, (**c**) Mean for BZDs among patients aged <75 years, (**d**) Mean for BZDs among patients aged ≥75 years, (**e**) Mean for Z-drugs among patients aged <75 years, (**f**) Mean for BZDs among patients aged ≥75 years.

Figure 3 shows the trends of the mean of average daily doses (diazepam-equivalent mg/day) for a patient in a month by the age groups and the type of drug. There results were relatively similar to those of the mean of number of drugs, and a decreasing trend was not observed for BZRAs regardless of the age groups from about 2016. For Z-drugs, an increasing trend was observed from about 2015 among patients aged <75 years, while a slight decreasing trend was observed in the periods among patients aged ≥75 years.

Table 2 shows the result of the MPC by segments for each outcome indicator, drug type, and age group. The proportion of BZRAs showed a large decreasing trend in the periods from December 2015 to April 2018 both among patients aged <75 and ≥ 75 years. For Z-drugs, the proportion turned to a significantly decreasing trend in April 2018 to March 2021 among the patients aged ≥75 years. The mean number of the types of drugs showed a significant decreasing trend in April 2009 to December 2015 segment among the patients aged <75 years and in April 2015 to April 2018 segment among the patients aged ≥75 years for BZRAs, and the trend was relatively similar to BZDs. However, an increasing trend was observed in the recent segment regardless of the type of drug, particularly among the patients aged ≥75 years. Regarding the mean average daily dose, a significant decrease was observed in April 2009 to December 2015, irrespective of the types of drugs and the age groups. However, a significant increasing trend was observed in recent years among patients aged <75 years.

## 4. Discussion

We revealed the prescription trend of BZRAs using the EHR data in a University Hospital in Japan. We found that the prescription trend differed with age group, type of drug, and the outcome indicators of the prescription trend. We discuss the possible reasons for these differences. The proportion of patients, in both age groups, who were prescribed BZRAs within those prescribed hypnotics or anxiolytics began to decrease particularly from 2015, and the degree of decrease was larger in those aged ≥75 years. One possible reason for this phenomenon is the effect of the Guidelines for Medical Treatment and its Safety in the Elderly edited in 2015 [8]. The guideline designates BZRAs as drugs that should be prescribed cautiously for elderly patients. Although BZDs are already designated as drugs that should be prescribed carefully for the elderly in the Guidelines for Medical Treatment and its Safety in the Elderly edited in 2005 [18], Z-drugs were newly added as drugs that also need to be prescribed carefully. Beers criteria, which were updated in 2012, newly listed Z-drugs as drugs that are to be prescribed cautiously [19], and the change probably affected the contents of the guideline in Japan. In fact, the proportion of patients prescribed Z-drugs within those prescribed hypnotics or anxiolytics began to decrease from 2016 in the elderly, which is considered to be related to the effect of the 2015 guidelines. The proportion for BZDs showed a decreasing trend from 2009 in the elderly patients and might be related to the effect of the 2005 guideline. In addition, the adverse effects of BZDs were already reported in the beginning of the analyzed periods, or earlier, not only against elderly patients [20,21,22], and considered to be related to the result of the proportion of BZDs use in patients aged <75 years. However, the proportion of Z-drugs users increased from 2009 until 2016 or 2017, irrespective of the patient age group, and this phenomenon could be related to the effect of the guidelines. The Clinical Practice Guideline for the proper use and cessation of hypnotics was published by The Japanese Society of Sleep Research in 2013 [23], and safety concerns of BZDs are mentioned in multiple parts of these guidelines. In addition, the Guideline of psychotropic drugs use against Behavioral and Psychological Symptoms of Dementia (BPSD) for primary care doctors was published in 2013 by the Ministry of Health, Labor, and Welfare [24], related to which the switch to Z-drugs should be considered when a patient already uses BZDs.

There was a decrease in the mean of number of the types of drugs prescribed to a patient in a month. The mean of number of the types of BZRAs particularly decreased from about 2014 for patients aged <75 years and patients aged ≥75 years. The decrease for BZDs was related to the decrease for BZRAs. Medical fee reduction in cases when three or more hypnotics or three or more anxiolytics were prescribed simultaneously was implemented in April 2014 in Japan [2]. Although the medical fee reduction for psychotropic drugs began in April 2012 [2], the degree of medical fee reduction largely increased in 2014. It is considered that the policy was effective for reducing the number of drugs prescribed to a patient. The results for the mean of average daily doses (diazepam-equivalent mg/day) for a patient in a month were relatively similar to those of the mean of the number of drugs. It is considered that the reduction in the number of drugs for a patient decreased the mean of the average daily doses for a patient.

The prescription trends for the mean number of the types of drugs for a patient and the average daily diazepam-equivalent doses were relatively stable from about 2015, irrespective of patient ages and type of drugs. Rather, an increase in the indicators has been observed in the previous few years. Therefore, based on the present results, the prescription amount for a patient using BZRAs did not show any reduction in recent years; however, the proportion of patients using BZRAs was continuously decreasing. The medical fee reduction for BZRAs was implemented in April 2018, and it is applied when same ingredients are prescribed by same doses per day continuously for >1 year. Although it is considered that the policy was implemented to prevent unnecessary long-term prescriptions of BZRAs, it is not applied even when diazepam-equivalent dosages per day are increased. Therefore, the effect of the policy might be limited for reducing the frequency of BZRAs prescription, and a method for reducing the daily diazepam-equivalent doses and frequency of BZRA prescriptions needs to be identified.

The degree of decrease in the proportions of patients prescribed BZRAs within those prescribed hypnotics or anxiolytics differed between patients aged <75 years and those aged ≥75 years, and it was suggested that some guidelines were responsible for these results. Therefore, it is considered that these guidelines are effective in changing the prescription trend, and publishing a method for reducing dosages and number of drugs for BZRAs in these guidelines would help in changing prescription trends. As methods for reducing prescription amount of BZRAs, psychological therapy or education, such as cognitive behavioral therapy are used [25,26,27], and a method of reducing BZRAs prescriptions in clinical practice is required. Otherwise, it will be effective to launch medical fee revisions concerning the prescribed number of BZRAs to reduce the number of BZRAs. In addition, it was suggested from this study that it might be meaningful to formulate some guidelines or medical fee revisions for unfavorable medical practices in order to engage in better medical practices and reduce burgeoning medical costs in Japan.

There are certain limitations of this study. First, the study used data from a University Hospital, and it is considered that there are cases where a patient is prescribed drugs at other hospitals. In addition, the prescription trends might differ based on the type of hospital. A study using nationwide claims data, such as the National Database of Health Insurance Claims and Specific Health Checkups in Japan [28,29] is warranted to investigate the prescription trends of BZRAs in Japan. Second, when calculating the average daily dose for a patient, we assumed that the drugs were taken every day from the date of first administration. However, the patient did not necessarily take the medicine every day or take all the prescribed medicines, and patient compliance data could not be obtained from the EHRs. Therefore, the average daily doses for a patient may have been overestimated in this study. Third, in some cases, BZRAs or other hypnotic and anxiolytics are necessary for medical treatment and are used for purposes such as sedation at the time of operation, and palliative care in cancer patients [30]. Hence, it might not necessarily be true that the lesser prescription amount of BZRAs, the better the medical practice, and further investigation of the prescription trend of BZRAs, considering the quality of medical care, is also needed. Fourth, we did not take into account disease information in the analysis. Additionally, it is possible that the characteristics of patients prescribed hypnotics or anxiolytics changed in the periods. By incorporating disease information, we could better understand the relationship between the trend of drug prescriptions and diseases [31]. However, one strength of this study is that we revealed the prescription trends, particularly in elderly patients, and trends observed in recent years. Although studies using large health insurance claim data have been conducted for investigating the prescription trends of BZRAs in Japan [1,10,11], to our knowledge none has investigated the prescription trends in elderly patients because few elderly persons have been analyzed. To our knowledge, this is the first study to investigate the long-term prescription trends of BZRAs in elderly people in Japan and is the first study to assess the trends in recent years. We believe that our results are useful for discussing future policies for psychotropic drugs, although a larger pharmacoepidemiologic study is necessary for verifying our study results.

## 5. Conclusions

We revealed the prescription trend of BZRAs using the EHRs data from a University Hospital in Japan. We found that the proportion of patients prescribed BZRAs among those prescribed hypnotics or anxiolytics began to decrease particularly from about 2015, for both patients aged <75 years and those aged ≥75 years. Moreover, the degree of decrease was larger in those aged ≥75 years. The proportion for BZDs decreased particularly during the study period, and the proportion for Z-drugs also began to decrease from 2016 in those aged ≥75 years. It was suggested that some guidelines for regulating the prescriptions of BZRAs, particularly among those aged ≥75 years, in the last decade affected the difference between the two age groups. In contrast, we found that the mean number of the types of drugs and average daily diazepam-equivalent doses of BZRAs for a patient did not decrease in recent years, irrespective of the patient age group, and a method for reducing the number of drugs and dosages among BZRAs users warrants further investigation. Moreover, a larger pharmacoepidemiologic study is necessary for verifying our study results.

## Figures and Tables

**Table 1:** Annual characteristics of the patients who were prescribed anxiolytics or hypnotics.

Characteristics	Year in the Japanese Calendar ^a^											
	2009	2010	2011	2012	2013	2014	2015	2016	2017	2018	2019	2020
Total (N)	11,235	11,149	10,731	14,862	10,119	10,093	9962	10,068	10,090	9972	9912	9497
Age group ^b^												
<75 years (%)	83.6	83.7	84.2	83.6	83.0	82.3	81.5	80.6	79.5	78.2	77.7	77.7
≥75 years (%)	17.0	17.0	16.4	16.8	17.6	18.2	19.4	20.4	21.4	22.6	23.1	22.9
Sex												
Men (%)	44.6	44.1	44.7	43.0	44.1	45.3	44.4	44.6	44.1	43.4	43.8	44.6
Women (%)	55.4	55.9	55.3	57.0	55.9	54.7	55.6	55.4	55.9	56.6	56.2	55.4
BZRAs (%)	98.7	98.9	98.4	98.1	98.1	97.3	96.7	94.3	88.5	84.6	79.2	72.1
BZDs (%)	85.8	85.4	82.1	80.6	78.9	77.5	75.6	71.9	66.2	62.4	56.9	50.3
Z-drugs (%)	32.9	33.3	36.6	35.3	39.6	39.4	40.9	41.2	37.9	36.8	35.8	34.2

^a^ Year in the Japanese calendar begins in April. For example, 2009 year begins April 2009 and ends in March 2010. ^b^ The sum of percentages of patients aged ≥75 years and <75 years does not become 100 because there are cases where a patient turns 75 years old in a year.

**Table 2:** The results of MPC by segments for each outcome indicator, drug type, and age group.

	Segment1	Segment2	Segment3
	April 2009–December 2015	December 2015–April 2018	April 2018–March 2021
Proportion ^a^			
<75 years			
BZRAs	−0.04 *	−0.26 *	−0.33 *
BZDs	−0.13 *	−0.30 *	−0.48 *
Z-drugs	0.09 *	−0.27 *	0.01
≥75 years			
BZRAs	−0.03 *	−0.41 *	−0.43 *
BZDs	−0.19 *	−0.71 *	−0.56 *
Z-drugs	0.37 *	0.00	−0.27 *
Mean number of the types of drugs ^b^			
<75 years			
BZRAs	−0.11 *	−0.08 *	0.06 *
BZDs	−0.09 *	−0.07 *	0.10 *
Z-drugs	0.00	−0.01	0.10 *
≥75 years			
BZRAs	0.01	−0.17 *	0.22 *
BZDs	0.00	−0.11	0.28 *
Z-drugs	0.06 *	−0.10	0.04
Mean of average daily doses ^c^			
<75 years			
BZRAs	−0.19 *	−0.02	0.17 *
BZDs	−0.13 *	0.00	0.24 *
Z-drugs	−0.23 *	−0.07	0.30 *
≥75 years			
BZRAs	−0.12 *	0.17	0.03
BZDs	−0.03	0.24 *	0.08
Z-drugs	−0.27 *	0.08	0.00

^a^ The proportion of patients prescribed with drugs among those prescribed hypnotics or anxiolytics in a month. ^b^ The mean number of the types of drugs prescribed to a patient in a month. ^c^ The mean average daily doses (diazepam-equivalent mg/day) for a patient in a month. * *p*-value < 0.05. MPC, Monthly percent change.

## Data Availability

The datasets analyzed during the current study are not publicly available because hospital data were used, but are available from the corresponding author on reasonable request.
